# Neuropeptide Y1 receptor antagonism protects β-cells and improves glycemic control in type 2 diabetes

**DOI:** 10.1016/j.molmet.2021.101413

**Published:** 2021-12-07

**Authors:** Chieh-Hsin Yang, Danise Ann-Onda, Xuzhu Lin, Stacey Fynch, Shaktypreya Nadarajah, Evan G. Pappas, Xin Liu, John W. Scott, Jonathan S. Oakhill, Sandra Galic, Yanchuan Shi, Alba Moreno-Asso, Cassandra Smith, Thomas Loudovaris, Itamar Levinger, Decio L. Eizirik, D. Ross Laybutt, Herbert Herzog, Helen E. Thomas, Kim Loh

**Affiliations:** 1 St. Vincent's Institute of Medical Research, Fitzroy, VIC, 3065, Australia; 2 Mary MacKillop Institute for Health Research, Australian Catholic University, Melbourne, VIC, 3000, Australia; 3 The Florey Institute of Neuroscience and Mental Health, Parkville, VIC, 3052, Australia; 4 Department of Medicine, University of Melbourne, Fitzroy, VIC, 3065, Australia; 5 Garvan Institute of Medical Research, St Vincent's Hospital, Sydney, 2010, Australia; 6 Faculty of Medicine, UNSW Australia, Sydney, 2052, Australia; 7 Institute of Health and Sport (IHES), Victoria University, Footscray, VIC, Australia; 8 Australian Institute for Musculoskeletal Science (AIMSS), University of Melbourne and Western Health, St Albans, VIC, Australia; 9 ULB Center for Diabetes Research, Medical Faculty, Universite Libre de Bruxelles (ULB), Brussels, Belgium; 10 Indiana Biosciences Research Institute (IBRI), Indianapolis, IN, USA

**Keywords:** NPY, β-Cell, Insulin secretion, Y1 receptor, Type 2 diabetes

## Abstract

**Objectives:**

Loss of functional β-cell mass is a key factor contributing to poor glycemic control in advanced type 2 diabetes (T2D). We have previously reported that the inhibition of the neuropeptide Y1 receptor improves the islet transplantation outcome in type 1 diabetes (T1D). The aim of this study was to identify the pathophysiological role of the neuropeptide Y (NPY) system in human T2D and further evaluate the therapeutic potential of using the Y1 receptor antagonist BIBO3304 to improve β-cell function and survival in T2D.

**Methods:**

The gene expression of the NPY system in human islets from nondiabetic subjects and subjects with T2D was determined and correlated with the stimulation index. The glucose-lowering and β-cell-protective effects of BIBO3304, a selective orally bioavailable Y1 receptor antagonist, in high-fat diet (HFD)/multiple low-dose streptozotocin (STZ)-induced and genetically obese (*db/db*) T2D mouse models were assessed.

**Results:**

In this study, we identified a more than 2-fold increase in *NPY1R* and its ligand, *NPY* mRNA expression in human islets from subjects with T2D, which was significantly associated with reduced insulin secretion. Consistently, the pharmacological inhibition of Y1 receptors by BIBO3304 significantly protected β cells from dysfunction and death under multiple diabetogenic conditions in islets. In a preclinical study, we demonstrated that the inhibition of Y1 receptors by BIBO3304 led to reduced adiposity and enhanced insulin action in the skeletal muscle. Importantly, the Y1 receptor antagonist BIBO3304 treatment also improved β-cell function and preserved functional β-cell mass, thereby resulting in better glycemic control in both HFD/multiple low-dose STZ-induced and *db/db* T2D mice.

**Conclusions:**

Our results revealed a novel causal link between increased islet NPY-Y1 receptor gene expression and β-cell dysfunction and failure in human T2D, contributing to the understanding of the pathophysiology of T2D. Furthermore, our results demonstrate that the inhibition of the Y1 receptor by BIBO3304 represents a potential β-cell-protective therapy for improving functional β-cell mass and glycemic control in T2D.

## Highlights

• Increased islet *NPY* and *NPY1R* levels are observed in human type 2 diabetes.
• Increased *NPY* and *NPY1R* expression is correlated with reduced beta-cell function.
• Inhibition of the Y1 receptor preserves functional beta-cell mass in type 2 diabetes.
• Inhibition of the Y1 receptor improves glycemic control in preclinical mouse models of type 2 diabetes.

## 1 Introduction

The prevalence of diabetes has been increasing over the last few decades, and currently, diabetes is a major health concern worldwide [1]. It is the major cause of premature mortality and other health complications such as cardiovascular disease and chronic kidney disease [2,3]. Located within the islets of Langerhans, pancreatic β-cells synthesize the hormone insulin, which is secreted primarily in response to elevated blood glucose levels. Type 2 diabetes (T2D) is the result of insufficient production of the glucose-lowering hormone insulin [4,5], triggered by multiple factors. Peripheral insulin resistance, coupled with diabetogenic stressors including hyperlipidemia, endoplasmic reticulum (ER), oxidative stresses, and inflammation are recognized as major driving forces of β-cell dysfunction and death, which ultimately leads to or exacerbates hyperglycemia, a key hallmark of T2D [[6], [7], [8]]. Although current treatments have proven to be successful in improving glycemic control in T2D patients, the existence of variations in the effectiveness of therapies and their tolerance among patients leads to a clinical need for new diabetic therapies that not only offer glucose-lowering effects but also address the underlying β-cell failure by preserving functional β-cell mass while improving insulin responsiveness.

The neuropeptide Y system consists of neuropeptide Y (NPY), peptide YY (PYY), and pancreatic polypeptide (PP), which are a group of short (36 amino acid) peptides that play a key role in the regulation of energy homeostasis [9,10]. NPY centrally promotes feeding and reduces energy expenditure, while PYY and PP mediate satiety [9,10]. The NPY system exerts its biological actions via a set of G-protein-coupled receptors (GPCR), of which five have been cloned: Y1, Y2, Y4, Y5, and Y6 [9,10]. The NPY system is widely expressed in the central nervous system as well as in peripheral tissues [9,10]. In the pancreas, while PYY and PP are expressed by α-cells and pancreatic PP cells, respectively, recent studies have demonstrated that NPY expression in mouse islet β-cells may play a role in altered β-cell function that precedes diabetes onset [10,11]. Interestingly, NPY levels were significantly upregulated in response to oxidative stress in islets from subjects with T2D [11]. Furthermore, NPY-deficient mice exhibit enhanced insulin secretion in response to glucose administration [12]. This is further confirmed by *in vitro* studies demonstrating that the application of NPY and PYY decrease glucose-stimulated insulin secretion from isolated mouse islets as well as from immortalized rodent BRIN BD11 and human 1.1B4 β cells [[12], [13], [14], [15]]. Together, these results suggest that NPY may act through a paracrine mechanism to tonically suppress β-cell function.

In addition to the brain, we previously identified that the neuropeptide Y1 receptor is also expressed in mouse and human β-cells and acts as a critical negative regulator of β-cell function [10,16,17]. Like all Y-receptors, the Y1 receptor is a GPCR that preferentially associates with Gi/o G-protein and therefore acts in an inhibitory manner in reducing cyclic AMP (cAMP) levels. Indeed, we have shown that pharmacological inhibition of this receptor using a Y1 receptor-specific antagonist, BIBO3304, significantly enhances β-cell function via a cAMP-dependent mechanism in mouse and human islets [16]. In addition, we have demonstrated that BIBO3304 delays the onset of type 1 diabetes (T1D) and may also be useful in boosting β-cell function under conditions where insulin secretion is limited such as during islet transplantation [16]. However, the beneficial effects of the pharmacological inhibition of the Y1 receptor in T2D remain unknown. Here, we show in proof-of-concept studies that the Y1 receptor antagonist BIBO3304 acts as a β-cell protective agent. BIBO3304 treatment significantly improved glycemic control in the HFD/multiple low-dose STZ-induced and obese leptin receptor-deficient (*db/db*) T2D mouse models. Importantly, our findings have direct relevance to the clinical setting of T2D, since BIBO3304 exhibited equal efficacy in improving glycemic control as the first-line oral antidiabetic drug, metformin.

## 2 Results

### 2.1 Increased NPY and Y1 receptor levels in T2D islets are associated with reduced insulin secretion

**Figure 1:**
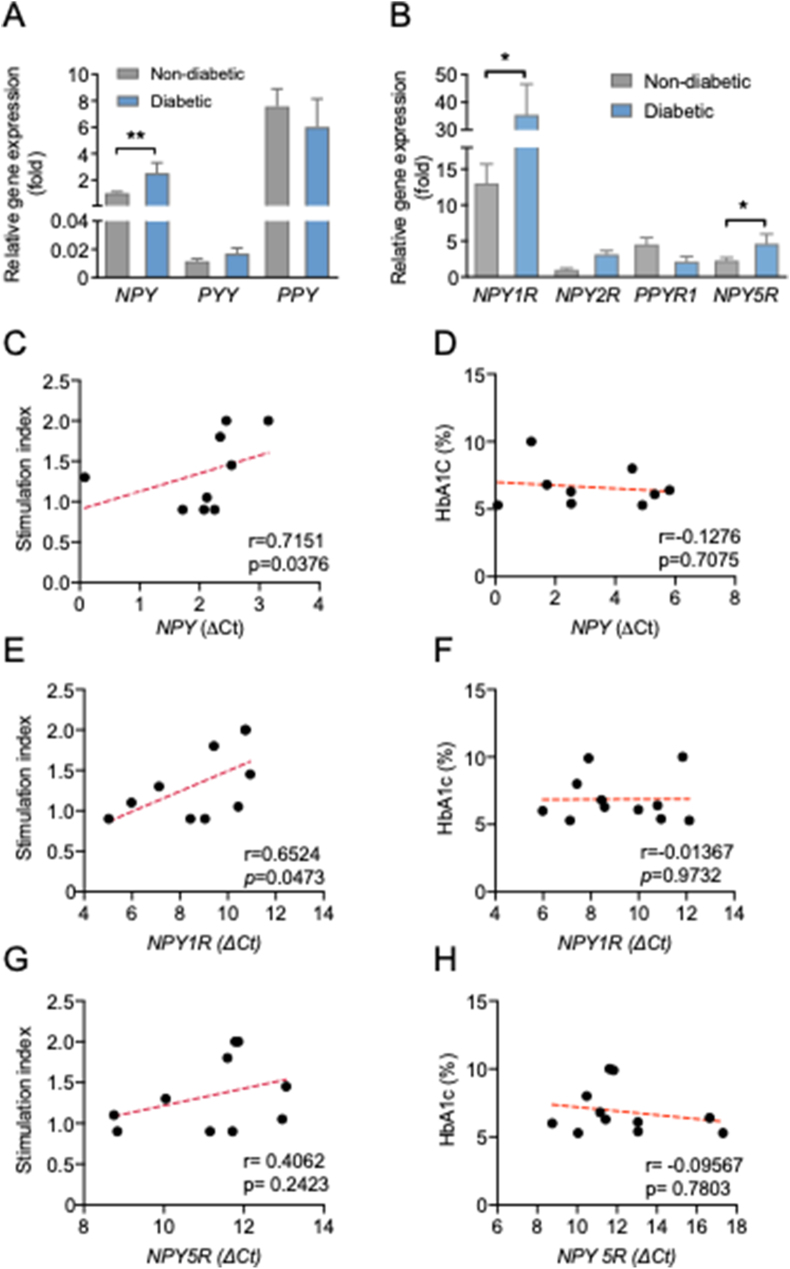
**Increased *NPY* and NPY1R mRNA expression levels are negatively correlated with the islet stimulation index in T2D.** (A) *NPY*, *PYY,* and *PPY* mRNA expression in human pancreatic islets from nondiabetic and T2D subjects relative to the *NPY* expression in the nondiabetic group. Subject numbers: nondiabetic = 25 and T2D = 11. (B) Y-receptor expression profiles in human pancreatic islets from nondiabetic and T2D subjects relative to the *NPY2R* expression in the nondiabetic group. Subject numbers: nondiabetic = 25 and T2D = 11. The results are normalized to the *RPLP0* gene (C–D). Correlation between the insulin stimulation index or HbA1C and the expression of *NPY* mRNA (delta CT values) in human islets of subjects with T2D and nondiabetic control subjects. Total subjects = 9. (E–F) Correlation between the insulin stimulation index or HbA1C and the expression of *NPY1R* mRNA (delta CT values) in human islets of subjects with T2D and nondiabetic control subjects. Total subjects = 10. (G–H) Correlation between the insulin stimulation index or HbA1C and the expression of *NPY5R* mRNA (delta CT values) in human islets of subjects with T2D and nondiabetic control subjects. Total subjects = 11. (A–B) Data are expressed as the mean ± SEM. ∗*P* < 0.05, ∗∗*P* < 0.01, calculated by the Student's t-test when comparing nondiabetic vs T2D subjects. (C–H) *P* values are derived from two-tailed Spearman correlation analysis.

**Supplemental Figure 1:**
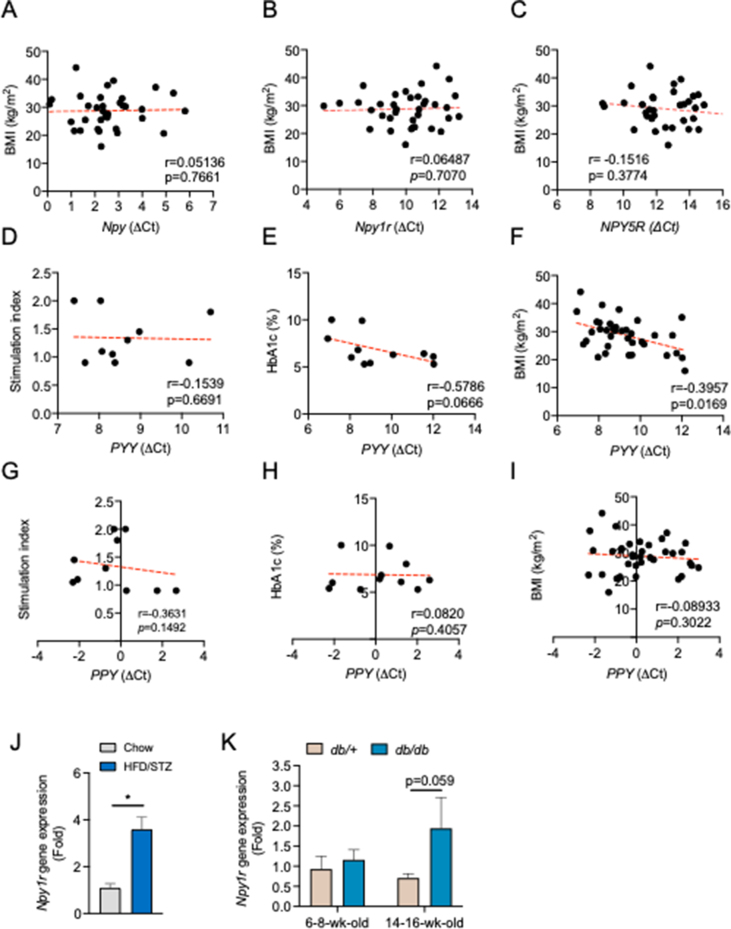
Correlation between the stimulation index, HbA1c or BMI and the islet NPY system expression in type 2 diabetes. (A–C) Correlation between the BMI and the expression of *NPY*, *NPY1R,* and *NPY5R* mRNA in human islets of subjects with type 2 diabetes and nondiabetic control subjects. Total subjects = 34. (D–F) Correlation between the insulin stimulation index, BMI, or HbA1C and the expression of *PYY* mRNA in human islets of subjects with type 2 diabetes and nondiabetic control subjects. Analysis was performed with a total number of 9 subjects for the stimulation index, 36 subjects for BMI, and 11 subjects for HbA1C. (G–I) The correlation between the insulin stimulation index, BMI, or HbA1C and the expression of *PPY* mRNA in human islets of subjects with type 2 diabetes and nondiabetic control subjects. Total number of 10 subjects in the stimulation index, 36 subjects in the BMI, and 11 subjects in HbA1C. Data are expressed as the mean ± SEM. *P* values are calculated using two-tailed Spearman's correlation analysis. (J) *Npy*, *Pyy,* and *Npy1r* mRNA expression in mouse islets isolated from HFD/STZ diabetic mice (*n* = 3) and age-matched C57BL/6 mice (*n* = 7). (K) *Npy1r* mRNA expression in young and aged *db/db* mouse islets compared with their age-matched *db/+* (*n* = 4–6) counterparts. The results are normalized to the *Ppia* gene. Data are expressed as the mean ± SEM. ∗*P* < 0.05, calculated by the Student's *t*-test when comparing nondiabetic (*db/+* or chow C57BL/6) vs diabetic (*db/db* or HFD/STZ) subjects.

To investigate whether the NPY system in pancreatic islets is associated with reduced β-cell function in the pathogenesis of T2D, we first profiled the NPY system gene expression in human islets isolated from nondiabetic and T2D subjects as described in Methods and Supplementary data. Our results revealed that in islets from diabetic donors, the expression of *NPY* and its receptor *NPY1R* was increased by 2.7- and 2.5-fold, respectively, compared with that observed in nondiabetic donors (Figure 1A and B). Importantly, the increase in *NPY* and *NPY1R* mRNA expression in human islets was accompanied by reduced insulin secretion as indicated by the insulin stimulation index (Spearman's r = 0.7151, *P* = 0.0376 and r = 0.6524, *P* = 0.0473) (Figure 1C and E). However, the differential expression of *NPY* and *NPY1R* was not associated with HbA1c (Figure 1D and F) or BMI (Figure S1A and B), which may be attributable to the fact that these T2D donors were receiving medical interventions to maintain blood glucose in check. Taken together, these results suggest that elevated NPY/Y1 receptor gene expression may contribute to the impaired insulin secretion observed in humans with T2D.

On the other hand, the basal mRNA levels of *NPY2R* and *PPYR1* (also known as *NPY4R* and *NPY5R*) were very low in pancreatic islets (Figure 1B), indicating that these Y receptors are unlikely to play a major role in mediating NPY function in human pancreatic islets. As Y1 and Y5 receptors share a common promoter region, *NPY5R* mRNA expression was also moderately upregulated in the islets of T2D subjects (Figure 1B), but there was no significant correlation between *NPY5R* expression and the insulin stimulation index, HbA1c (Figure 1G and H) or BMI (Figure S1C). Importantly, the NPY/Y1 receptor axis appeared to be exclusively upregulated as there were no noticeable changes in other NPY ligands such as *PYY* and *PPY* and their correlation with the insulin stimulation index or HbA1c (Figure S1D–I). In concordance with the observations in human islets from T2D, we observed a significant increase in islet *Npy1r* mRNA levels from HFD/multiple low-dose/STZ-induced T2D mice (Figure S1J). While islet *Npy1r* gene expression was unaltered during β-cell compensation in young (6–8 weeks old) *db/db* mice, we found that islet *Npy1r* levels were upregulated in the β-cell decompensation stage, as observed in overt diabetic *db/db* mice (Figure S1K). Collectively, these results reveal a novel link between increased islet NPY/Y1 receptor signaling and β-cell dysfunction in human T2D.

### 2.2 Y1 receptor inhibition restores β-cell function and protects against β-cell apoptosis under diabetogenic conditions

**Figure 2:**
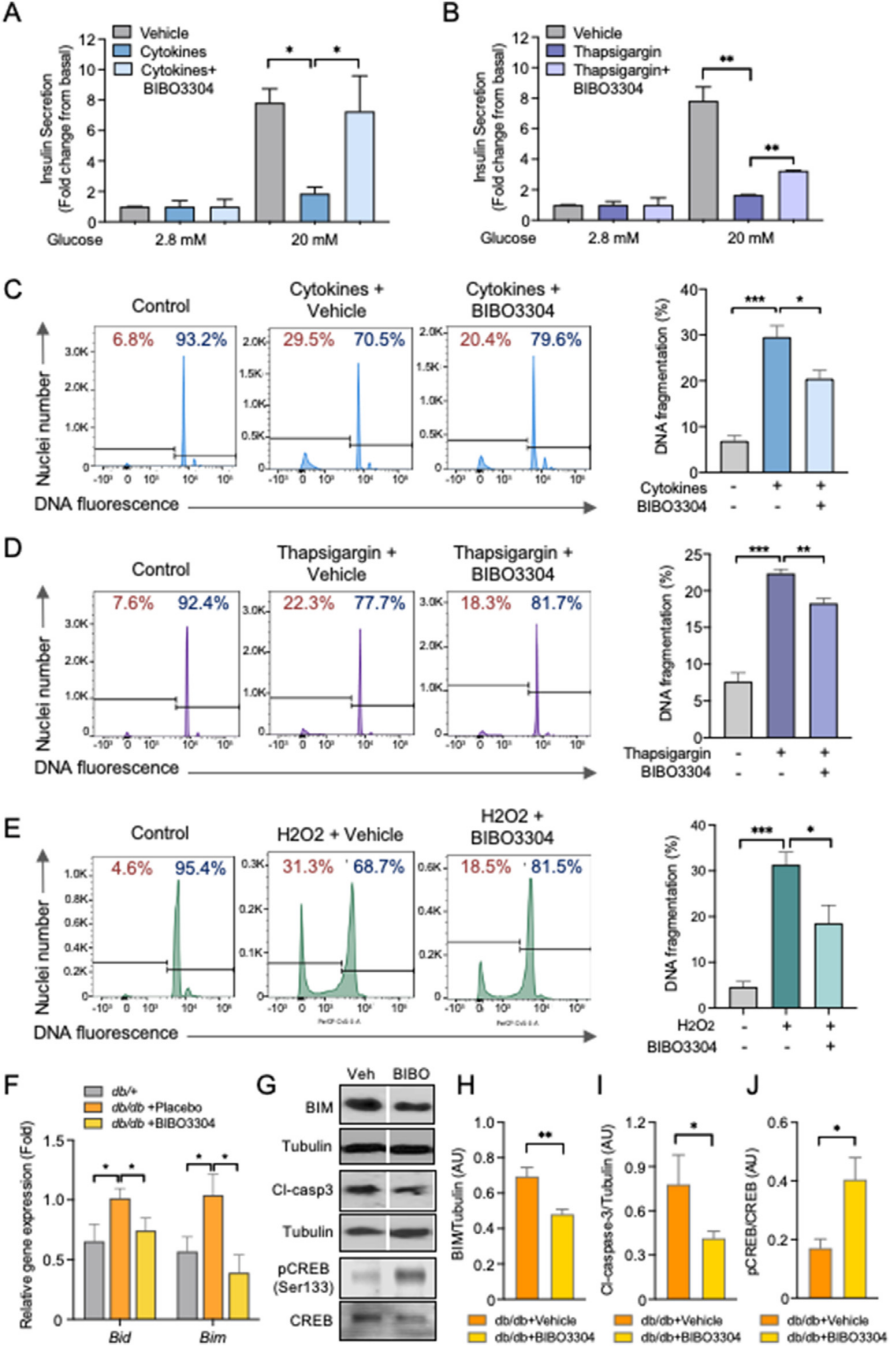
**Pharmacological inhibition of the Y1 receptor restores β-cell function and protects against β-cell death under diabetogenic conditions.** (A–B) Pancreatic islets from C57BL/6 mice were isolated and cultured under the corresponding diabetogenic conditions: inflammatory cytokine cocktail of 25 ng/ml IL-1β, 250 ng/ml IFNγ, 50 ng/ml TNFα ± 1 μM of BIBO3304 for 48 h (*n* = 5), thapsigargin (1 μM) ± 1 μM of BIBO3304 for 24 h (*n* = 3–6). Glucose-stimulated insulin secretion was determined in response to 2.8 and 20 mmol/L glucose. (C–E) DNA fragmentation in response to inflammation, ER stress, and oxidative stress was measured by flow cytometry. Representative FACS profiles are shown and the results are representative of islets from a minimum of 3 mice per group. (F) Apoptotic gene expression in islets from 14 to 16-week-old diabetic *db/db* mice treated with 1 μM BIBO3304 or placebo for 36 h. Data are expressed as the mean ± SEM of 4–6 mice. (G–J) Western blot analyses of pro-apoptotic proteins BIM, cleaved caspase-3, and phosphorylated CREB (Ser133) in isolated islets from 10 to 12-week-old leptin receptor-deficient *db/db* mice were cultured with/without 1 μM of BIBO3304 for 36 h. α-tubulin was used as the loading control (*n* = 3–4). The results shown are a representative blot and quantitative densitometry analysis. Data are expressed as the mean ± SEM. ∗*P* < 0.05, ∗∗*P* < 0.01, and ∗∗∗*P* < 0.001, calculated by the unpaired Student's *t*-test.

**Supplemental Figure 2:**
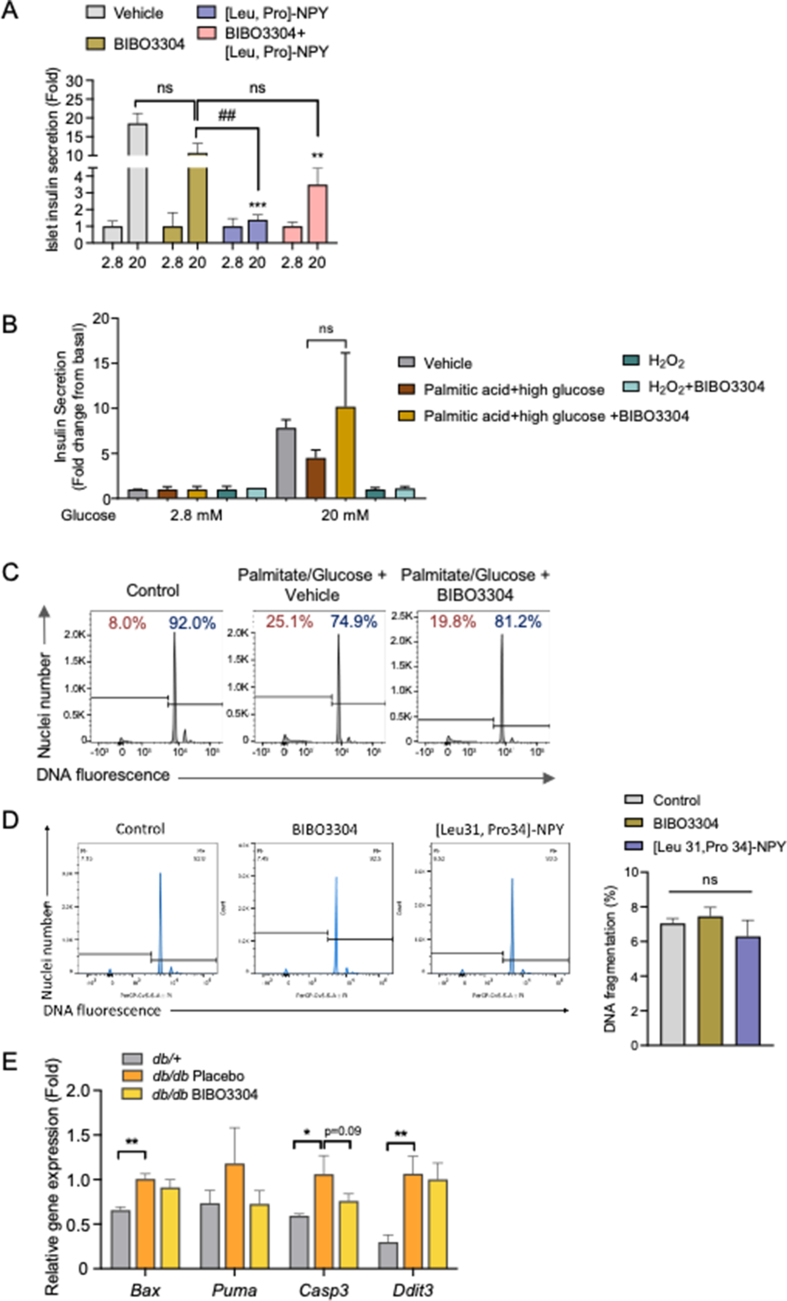
Islet cell death analysis and glucose-stimulated insulin secretion in Y1 receptor antagonist-treated islets under basal and glucolipotoxicity and oxidative stress conditions. (A) Pancreatic islets from C57BL/6 mice were isolated and cultured in the absence of diabetogenic stress or (B) were exposed to 25 mmol/L glucose and 0.5 mM palmitate ± 1 μM of BIBO3304 for 96 h or 10 μM H_2_O_2_ ± 1 μM of BIBO3304 for 16 h (*n* = 3–4). Glucose-stimulated insulin secretion was determined in response to 2.8 and 20 mmol/L glucose. (C) DNA fragmentation in response to glucolipotoxicity (25 mmol/L glucose and 0.5 mM palmitate ± 1 μM of BIBO3304 for 72 h) or (D) in the absence of diabetogenic stress was measured by flow cytometry. Representative FACS profiles are shown, and the results are representative of islets from a minimum of *n* = 3 per group. (E) Apoptotic gene expression in isolated islets from 14–16-week-old diabetic *db/db* mice treated with 1 μM BIBO3304 or placebo for 36 h. Data are expressed as the mean ± SEM of 4–6 mice. Data are expressed as the mean ± SEM. ∗*P* < 0.05 and ∗∗*P* < 0.01, calculated by the unpaired Student's *t*-test.

Diabetogenic stresses such as inflammation, ER stress, oxidative stress, and glucolipotoxicity have been implicated as key factors contributing to the impaired β-cell function and death in T2D [[18], [19], [20]]. Importantly, previous studies have shown that NPY-Y1 signaling in β cells was upregulated under diabetogenic stress conditions including oxidative stress [11], ER stress [28], and inflammation [29] in both mouse and human islets. Although our group and others have previously reported that activation of Y1 receptors by [Leu^31^, Pro^34^] NPY and PYY (3–36) suppresses insulin secretion, we further showed that this inhibition was restored in the presence of the Y1 receptor-specific antagonist BIBO3304 (Figure S2A). Given that islet NPY/Y1 receptor levels are negatively correlated with the insulin stimulation index in human T2D, we next investigated whether pharmacological inhibition of NPY/Y1 receptor signaling by a selective Y1 receptor antagonist BIBO3304, under diabetogenic conditions, would restore β-cell function. Selectivity of BIBO3304 for the Y1 receptor has previously been validated by our group and others using various receptor knockout cells [16,21,22]. To test this, we assessed glucose-stimulated insulin secretion (GSIS) treated with or without BIBO3304 on wild-type C57BL/6 islets that were exposed to various stress conditions (inflammation: proinflammatory cytokines TNFα, IFNγ, and IL1β; ER stress: thapsigargin; oxidative stress: H_2_O_2_; glucolipotoxicity: high glucose/palmitate). A significant reduction in insulin release in response to glucose stimulation was observed in islets that were exposed to inflammatory cytokines (Figure 2A) and ER stress (Figure 2B). In contrast, co-treatment with BIBO3304 prevented the impaired insulin release that was induced by proinflammatory cytokines or ER stress inducer, thapsigargin (Figure 2A and B), with no significant difference observed in the high-glucose/palmitate-treated islets (Figure S2B). Taken together, these results demonstrate that, under diabetogenic stress conditions such as inflammation and ER stress, Y1 receptor antagonism can directly restore β-cell function by enhancing GSIS.

Targeting β-cell preservation is a key component of the therapeutic strategies for glycemic control in diabetes [23]. We next investigated whether the inhibition of the Y1 receptor could also protect β-cells from failure under diabetogenic conditions by exposing islets to various stress conditions with or without the treatment of the Y1 receptor antagonist BIBO3304. As expected, chronic exposure to all diabetogenic stressors induced a substantial increase in β-cell apoptosis (Figure 2C–E). In contrast, BIBO3304 significantly reduced cytokine-, thapsigargin-, and H_2_O_2_-induced cell death (Figure 2C–E) but not glucolipotoxicity-induced cell death (Figure S2C) or in the absence of diabetogenic stress (Figure S2D), as indicated by the decreased subdiploid DNA content. Given that the pathogenesis of T2D is the result of complex metabolic perturbations, islets cultured with individual diabetogenic stress alone may have a limited ability to model the islet microenvironment in T2D. We therefore tested the ability of BIBO3304 to protect β-cells from death in islets isolated from severely diabetic *db/db* mice (random blood glucose (RBG) 27.5 ± 1.84 mmol/L; body weight (BW) 42.4 ± 1.56 g) compared with their nondiabetic littermate control *db/+* mice (RBG 10.5 ± 0.4 mmol/L; BW 24.5 ± 1.16 g). Analysis of gene expression revealed significant upregulation of apoptotic signaling in islets from 14 to 16-week-old overt diabetic *db/db* mice compared with *db/+* mice. Interestingly, BIBO3304 treatment significantly reduced cell death in *db/db* islets as demonstrated by the decrease in pro-apoptotic *Bid* and *Bim* expression (Figures 2F and S2E). In agreement with this, our results showed that the addition of BIBO3304 alleviated β-cell apoptosis in *db/db* islets as evidenced by the significant reduction in the expression of pro-apoptotic markers BIM and cleaved-Caspase-3 (Figure 2G–I). We have previously shown that phosphorylated cAMP responsive element-binding protein (pCREB) levels were significantly upregulated in islets of Y1 receptor-deficient or BIBO3304-treated WT islets [16]. Consistent with the effect of increased pCREB in promoting β-cell survival [24], we found that pCREB levels were also significantly upregulated by 2-fold in BIBO3304-treated *db/db* islets (Figure 2G and J). Taken together, these results suggest that the direct inhibition of Y1 receptor signaling in islets was associated with reduced apoptosis, indicating that Y1 receptor antagonism plays a protective role in preserving β-cell mass and function in T2D.

### 2.3 Inhibition of the Y1 receptor restores normoglycemia in HFD and STZ-induced T2D mouse models

**Figure 3:**
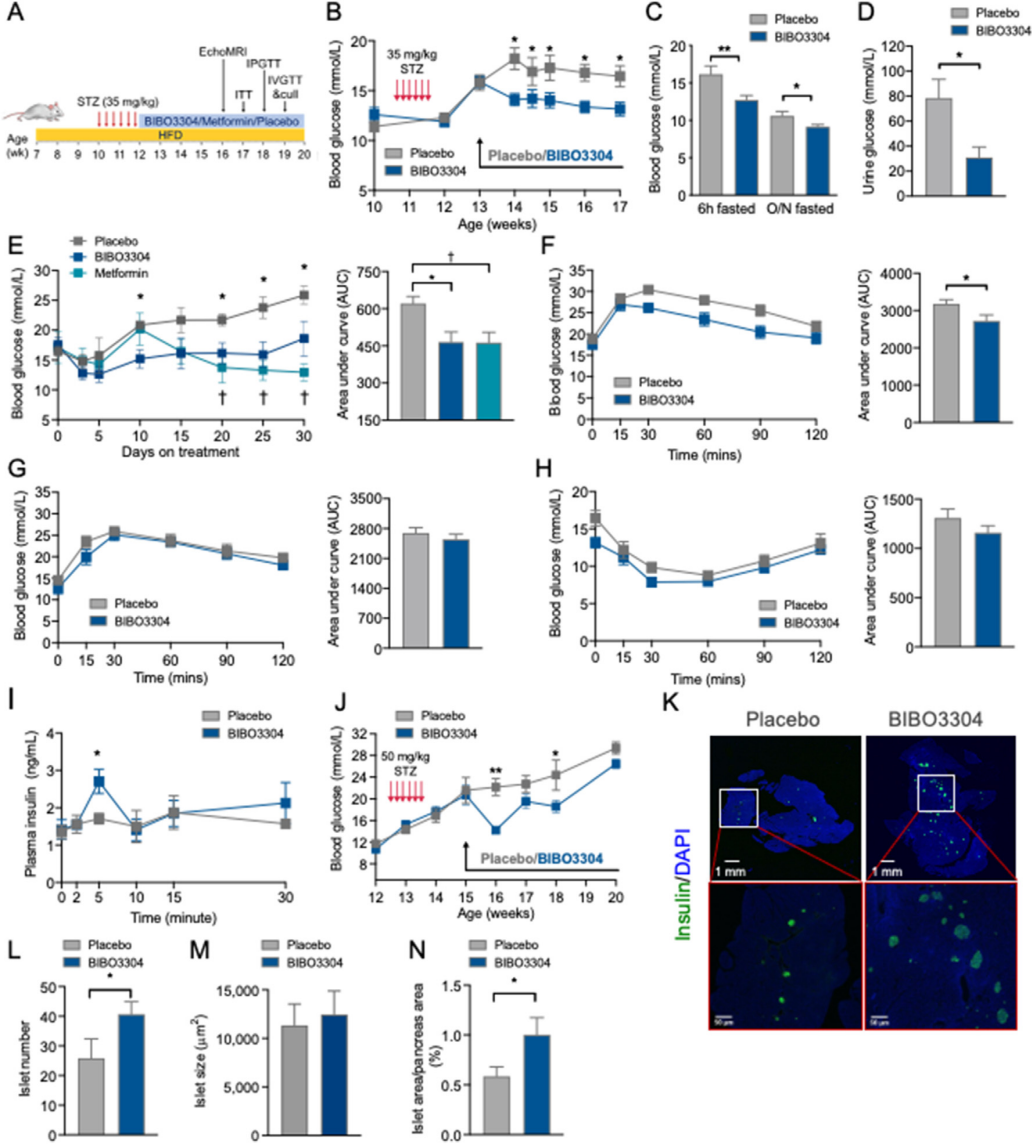
**Y1 receptor antagonist BIBO3304 improves glycemia in HFD/STZ-induced diabetic mice.** (A) Schematic diagram of the treatment regimen. C57BL/6 mice were fed on a high-fat diet for 4 weeks and rendered diabetic by multiple low doses of STZ injections (6 doses, 35 mg/kg). Diabetic mice were randomized to receive placebo, oral Y1 antagonist BIBO3304, or metformin for 6 weeks. Metabolic and glucose homeostasis parameters were examined thereafter. (B) Non-fasting blood glucose levels at the indicated time points were measured from placebo and BIBO3304-treated mice. *n* = 8 per group. (C) Six-hour and overnight-fasted blood glucose levels. *n* = 7–8 per group. (D) Urine glucose levels. *n* = 6–7 per group. (E) Non-fasting blood glucose levels at the indicated time points were measured from placebo, BIBO3304-treated mice, or metformin-treated mice. The results expressed as area under the curve. *n* = 4–6 per group. (F) Intraperitoneal glucose tolerance tests (1 g/kg body weight) on 6-h-fasted diabetic mice treated with placebo or BIBO3304 for 4 weeks. Blood glucose levels during glucose tolerance tests were monitored, and the results are expressed over the time course and as the area under the curve. *n* = 8 per group. (G–H) Diabetic mice treated with placebo or BIBO3304 were fasted overnight or for 6 h and i.p. pyruvate tolerance tests (1 g/kg body weight) or insulin sensitivity tests (0.75 i.u./kg body weight) were performed, respectively. Blood glucose levels during the tolerance tests were monitored, and the results are expressed over the time course and as the area under the curve. *n* = 6–8 per group. (I) Plasma insulin levels throughout intravenous glucose tolerance tests (1 g/kg body weight) from mice treated with placebo or BIBO3304. *n* = 5–6 per group. (J) C57BL/6 mice were rendered diabetic by multiple high doses of STZ injections (6 doses, 50 mg/kg body weight). Non-fasting blood glucose levels at the indicated time points were measured from placebo and BIBO3304-treated mice. *n* = 5–6 per group. (K) Sections of pancreas from placebo or BIBO3304-treated mice were stained for insulin (green) and nuclear counterstained with DAPI (blue). (L–N) Islet number, islet size, and islet proportion were determined across three nonconsecutive pancreatic sections per mouse and normalized to the total pancreatic section area. *n* = 5–6 per group. Data are expressed as the mean ± SEM. ∗*P* < 0.05 and ∗∗*P* < 0.01, calculated by the unpaired Student's *t*-test or two-way ANOVA analysis.

**Supplemental Figure 3:**
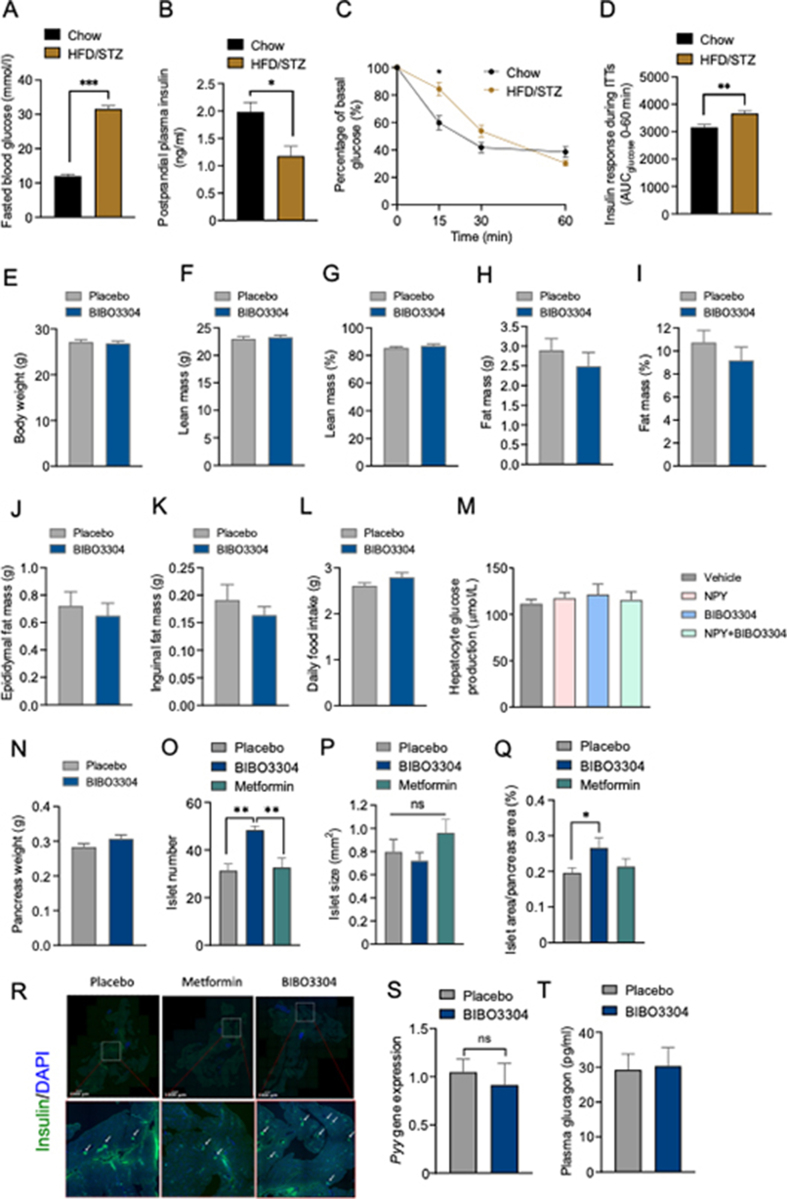
Y1 receptor antagonist BIBO3304 treatment did not alter adiposity, food intake, or hepatic glucose production in HFD/STZ-induced diabetic mice. C57BL/6 mice were fed a high-fat diet for 4 weeks and rendered diabetic by multiple low doses of STZ injections (6 doses, 35 mg/kg). Diabetic HFD/STZ mice exhibited (A) increased fasting blood glucose levels, (B) decreased postprandial insulin secretion, (C–D) impaired insulin response when compared with chow-fed C57BL/6 mice. (E–L) Diabetic mice were randomized to receive placebo or oral Y1 antagonist BIBO3304 for 4 weeks. Metabolic and glucose homeostasis parameters were examined thereafter. (E) Body weight of HFD/STZ-induced diabetic mice treated with placebo or oral BIBO3304 (*n* = 6–8 per group). (F–I) Whole-body lean and fat mass as determined by EchoMRI analysis in placebo or oral BIBO3304-treated HF/STZ mice (*n* = 6–8 per group). (J–K) Dissected weights of individual epididymal (Epi) and inguinal (Ing) white adipose tissue (*n* = 6–8 per group). (L) Daily food intake of HFD/STZ-induced diabetic mice treated with placebo or oral BIBO3304 (*n* = 6–8 per group). (M) Hepatocytes were isolated and glucose production was performed in the presence of NPY, BIBO3304, or NPY + BIBO3304 (*n* = 6–8 per group). (N) Dissected weights of pancreas from HFD/STZ-induced diabetic mice treated with placebo or oral BIBO3304 (*n* = 6–8 per group). (O–Q) Islet number, islet size, and islet proportion were determined across three nonconsecutive pancreatic sections per mouse from BIBO3304-, metformin-, and placebo-treated groups. *n* = 5–6 per group. (R) Images shown are representative of the pancreatic sections from placebo-, BIBO3304-, or metformin-treated mice stained for insulin (green) and DAPI (blue) for nucleus. (S) Islet *Pyy* gene expression of HFD/STZ-induced diabetes mice treated with placebo or oral BIBO3304 (*n* = 6–7 per group). (T) Plasma glucagon levels from HFD/STZ-induced diabetes mice treated with placebo or oral BIBO3304 (*n* = 8 per group). Data are expressed as the mean ± SEM. ∗*P* < 0.05 and ∗∗*P* < 0.01, calculated by the unpaired Student's *t*-test or one-way ANOVA.

While pharmacological inhibition of the Y1 receptor restored β-cell function and decreased β-cell death *ex vivo*, we next investigated whether BIBO3304 could improve glycemic control *in vivo* in the context of a nongenetic animal model of human adult-onset T2D that exhibits hyperglycemia and β-cell dysfunction. To test this, C57BL/6 mice were rendered diabetic by HFD diet feeding for 4 weeks followed by injection with multiple low-dose STZ to induce partial β-cell loss as demonstrated by significant increased 6-h-fasted blood glucose (31.65 ± 0.87 mmol/L vs 11.98 ± 0.53 mmol/L in age-matched C57BL/6) with impaired insulin responsiveness and postprandial insulin secretion (1.17 ± 0.17 ng/ml vs 1.98 ± 0.17 ng/ml in age-matched C57BL/6) (Figure S3A–D). Diabetic HFD/STZ mice were subsequently treated with placebo or BIBO3304 for up to 6 weeks in order to assess the ability of BIBO3304 to restore normoglycemia (Figure 3A). Significant hyperglycemia (blood glucose > 15 mmol/L) was established 7 days after STZ treatment (Figure 3B). Body weight, adiposity, and food intake were comparable between the BIBO3304- and placebo-treated groups (Figure S3E–L). Importantly, BIBO3304-treated mice exhibited significantly lower blood glucose levels during the entire 4 weeks of the study when compared with the placebo group (Figure 3B). Fed and fasting blood glucose levels were also significantly reduced in BIBO3304-treated mice (Figure 3C). The reduced blood glucose levels were unlikely due to an increase in urinary glucose excretion as urine glucose was 2–3-fold lower in BIBO3304-treated mice than in the placebo group (Figure 3D). To compare the effects of BIBO3304 with a currently available oral anti-diabetic drug, we tested the effects of metformin, the first-line and most widely prescribed drug for the treatment of T2D. Metformin treatment resulted in improved blood glucose levels in HFD/STZ diabetic mice (Figure 3E) and did not show significantly different results compared with the improvement in glycemic control by BIBO3304 (Figure 3E).

Consistent with the improved glycemic control, mice receiving BIBO3304 exhibited significantly improved glucose tolerance due to enhanced *in vivo* insulin secretory capacity (Figure 3F and I). To further determine whether the improved glycemic control observed in the BIBO3304-treated group was β-cell dependent, we subsequently examined the effects of BIBO3304 in mice with substantial β-cell loss by administering a higher dose of STZ (50 mg/kg). Interestingly, the improvement in glycemic control was no longer sustained after the first week of BIBO3304 treatment (Figure 3J), indicating that the glucose-lowering effect of the Y1 receptor antagonist BIBO3304 is dependent on its action on the functional β-cell mass. On the other hand, BIBO3304 treatment had no influence on hepatic glucose production or insulin responsiveness *in vivo* or *ex vivo* (Figures 3G, H, and S3M). In addition, there was a significant increase in islet numbers in pancreas from HFD/STZ mice receiving 4 weeks of BIBO3304 treatment (Figure 3K and L), while the pancreatic weights (Figure S3N) remained comparable. Despite islets from BIBO3304-treated mice being comparable in size to the placebo-treated group, the total islet area was significantly greater in the BIBO3304-treated mice, due to an increase in the islet number (Figure 3M and N). In contrast, the islet number, size, and proportion were comparable between diabetic mice treated with metformin and the placebo group (Figure S3O–R). Similarly, no noticeable changes in the islet *Pyy* gene expression or circulating glucagon levels between BIBO3304 and the placebo group were observed, indicating that Y1 receptor inhibition has no effect on the islet α-cell function (Figure S3S and T). Collectively, these results suggest that Y1 receptor antagonism may be clinically beneficial in improving glycemic control in T2D by enhancing functional β-cell mass.

### 2.4 Y1 receptor antagonism improves insulin responsiveness and β-cell function at various stages of diabetes progression

**Figure 4:**
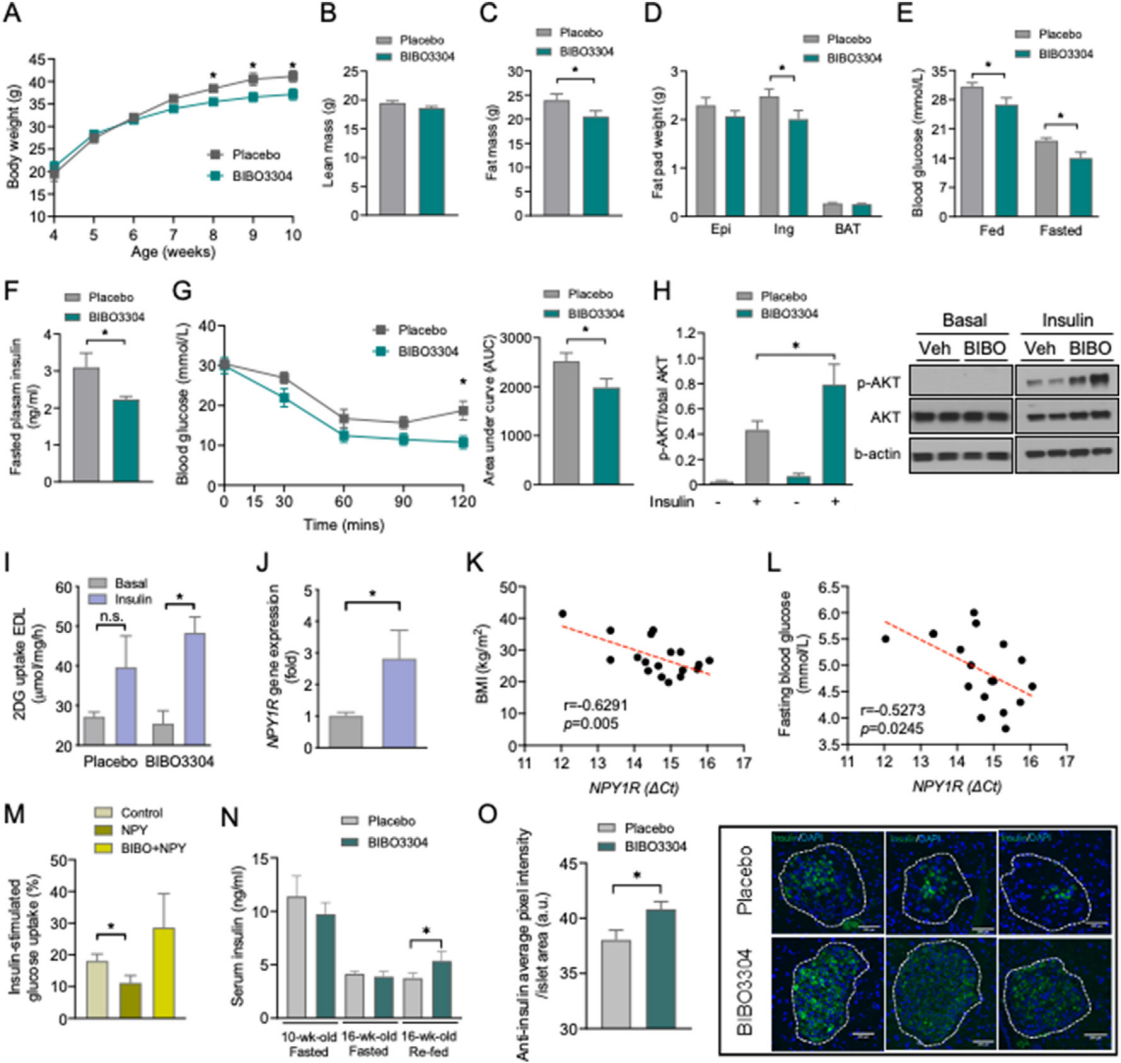
**Y1 receptor antagonist BIBO3304 improves hyperglycemia and insulin sensitivity and preserves functional β-cell mass in *db/db* mice.** Four-week-old leptin receptor-deficient *db/db* mice were randomized to receive placebo or oral Y1 antagonist BIBO3304 for 6 weeks. (A) Weekly body weight of *db/db* mice treated with placebo or oral BIBO3304 (*n* = 5–6 per group). (B–C) Whole-body lean and fat mass as determined by EchoMRI analysis in *db/db* mice treated with placebo or oral BIBO3304 (*n* = 7 per group). (D) Dissected weights of individual white adipose tissue from epididymal (Epi), inguinal (Ing), and brown adipose tissue (BAT) (*n* = 4–5 per group). (E) Fed and fasted blood glucose levels in *db/db* mice treated with placebo or oral BIBO3304 (*n* = 5–6 per group). (F) Fasting plasma insulin levels in *db/db* mice treated with placebo or oral BIBO3304 (*n* = 6–8 per group). (G) *db/db* mice treated with placebo or BIBO3304 were fasted for 6 h or overnight and intraperitoneal insulin tolerance tests (2.5 i.u./kg body weight) were performed. Blood glucose levels during the tolerance tests were monitored, and the results are expressed over the time course and as the area under the curve (*n* = 5 per group). (H–I) EDL muscle isolated from *db/db* mice treated with placebo or BIBO3304, and insulin-stimulated glucose uptake and Akt activation were determined. The muscle homogenates were subjected to SDS-PAGE and Western blot analysis using anti-phospho Ser 473 Akt, total Akt, and β-actin antibodies (*n* = 5–6). The results shown are a representative blot and quantitative densitometry analysis. The cropped gel is used in the figure and full-length gel is presented in Supplemental Figure S4C. (J) *NPY1R* expression in human muscle from lean (BMI < 25) and overweight/obese (BMI > 25) subjects. Subject numbers: lean = 7 and overweight/obese = 11. (K–L) Correlation between the fasting blood glucose or BMI and the expression of *NPY1R* mRNA (delta CT values) in human muscle of obese and lean control subjects. Total subjects = 18. (M) Primary human muscle cells (*n* = 3) were cultured and insulin-stimulated glucose uptake was determined following the treatment with NPY (Leu^31^, Pro^34^) or NPY + Y1 receptor antagonist BIBO3304. The results were presented as the percentage increase from basal, and the data are the average of 3 independent experiments. (N) Four- and ten-week-old leptin receptor-deficient *db/db* mice were randomized to receive placebo or oral Y1 antagonist BIBO3304 for 6 weeks. Fasted and re-fed serum insulin levels were measured (*n* = 5–8 per group). (O) Pancreases from placebo or BIBO3304-treated mice at 16 weeks of age were weighed and fixed in formalin and processed for immunostaining of insulin (green) and nuclear counterstained with DAPI (blue). Insulin intensity was determined by screening 138 and 172 islets on placebo and BIBO3304-treated pancreatic sections, respectively. Insulin intensity was presented as insulin positive pixel normalized to the islet area. Data are expressed as the mean ± SEM. ∗*P* < 0.05 calculated by the unpaired Student's *t*-test or two-way ANOVA analysis.

**Supplemental Figure 4:**
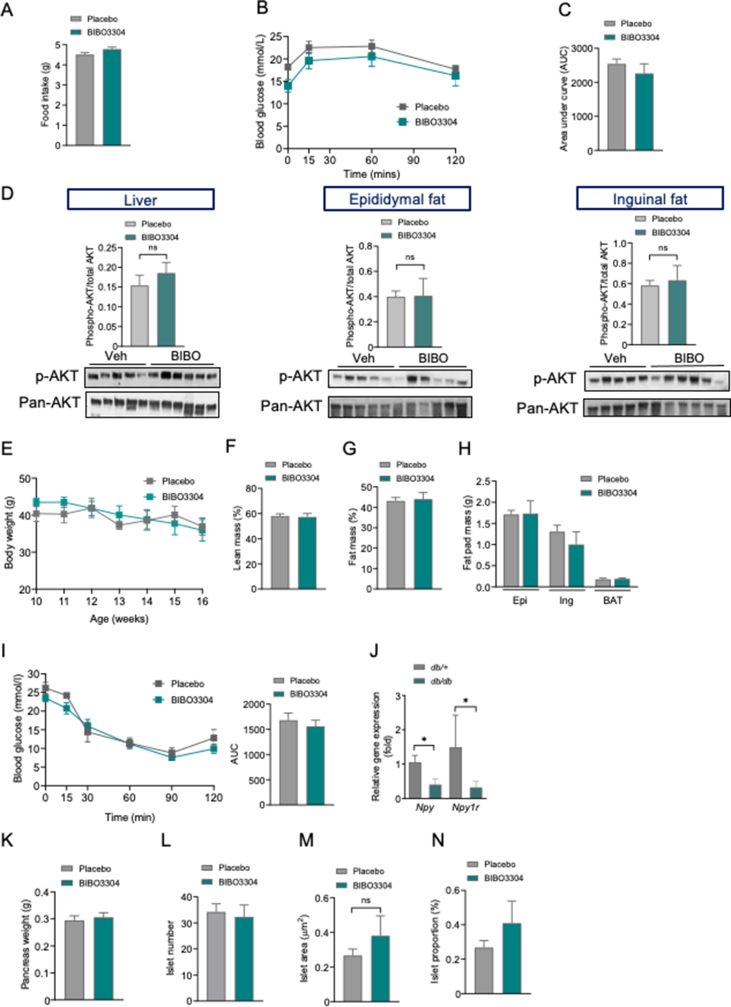
Metabolic and glucose homeostasis parameters in *db/db* mice treated with BIBO3304. Four-week-old leptin receptor-deficient *db/db* mice were randomized to receive placebo or oral Y1 antagonist BIBO3304 for 6 weeks. (A) Daily food intake of *db/db* mice treated with placebo or oral BIBO3304 (*n* = 5–6 per group). (B–C) *db/db* mice treated with placebo or BIBO3304 were fasted overnight, and intraperitoneal glucose tolerance tests (0.5 g/kg body weight) were performed. Blood glucose levels during the tolerance tests were monitored, and the results are expressed over the time course and as the area under the curve (*n* = 5–6 per group). (D) Livers and white adipose tissues were isolated from 10-week-old *db/db* mice treated with placebo or BIBO3304 and Akt activation was determined. Tissues were subjected to SDS-PAGE and Western blot analysis using anti-Akt phosphorylation Ser 473, and β-actin antibodies (*n* = 5–6). The results shown are a representative blot and quantitative densitometry analysis. (E–G) Ten-week-old leptin receptor-deficient *db/db* mice were randomized to receive placebo or oral Y1 antagonist BIBO3304 for 6 weeks. Weekly body weights were determined. Whole-body lean and fat mass was determined by EchoMRI analysis in placebo- or oral BIBO3304-treated *db/db* mice (*n* = 5 per group). (H) Dissected weights of individual epididymal (Epi) and inguinal (Ing) white adipose tissue and brown adipose tissue (BAT) (*n* = 4–5 per group). (I) *db/db* mice treated with placebo or BIBO3304 were fasted overnight, and intraperitoneal insulin tolerance tests (2.5 i.u./kg body weight) were performed. Blood glucose levels during the tolerance tests were monitored, and the results are expressed over the time course and as area under the curve. (*n* = 4–6 per group). (J–M) Pancreases from placebo- or BIBO3304-treated mice were weighed and fixed in formalin and processed for immunostaining of insulin (green) and nuclear counterstained with DAPI (blue). Islet number, islet area, and islet proportion were determined across two nonconsecutive pancreatic sections per mouse and normalized to the total pancreatic section area. *n* = 4–6 per group. Data are expressed as the mean ± SEM. ∗*P* < 0.05 and ∗∗*P* < 0.01, calculated by unpaired Student's *t*-test or two-way ANOVA analysis.

Genetically, diabetic leptin receptor-deficient *db/db* mice are obese and insulin resistant and display hyperglycemia at an early age and transition from β-cell compensation to failure with a pathophysiological sequence of events similar to human T2D [16]. The effects of BIBO3304 on glycemic control were also examined in *db/db* mice in the early (4–10 weeks old) and a late (10–16 weeks old) stages of T2D. Four-week-old *db/db* mice were treated daily with BIBO3304 or placebo over a period of 6 weeks. Interestingly, after 6 weeks of treatment, the BIBO3304-treated group showed a significantly lower body weight compared with placebo (Figure 4A). While lean mass remained comparable, the observed reduction in body weight in the BIBO3304-treated group was mostly due to a decrease in fat mass (Figure 4B and C). Consistently, the absolute weights of individual fat pads revealed that inguinal fat mass was significantly lower in the BIBO3304-treated group compared with placebo (Figure 4D). The reduction in body weight observed in the BIBO3304-treated group was not due to changes in appetite, as evidenced by the absence of a significant difference in food intake (Figure S4A). Importantly, fed and fasting blood glucose levels were significantly lower in BIBO3304-treated mice compared with placebo (Figure 4E). BIBO3304-treated mice also exhibited significantly lower fasting plasma insulin levels (Figure 4F), which is suggestive of an increase in insulin sensitivity. In agreement with this notion, insulin tolerance tests revealed that BIBO3304-treated *db/db* mice exhibited a markedly improved insulin responsiveness, as evidenced by lower blood glucose across the duration of 120 min and when quantified as area under the curve (Figure 4G). BIBO3304-treated mice displayed no improvement in whole-body glucose tolerance (Figure. S4B and C). The enhanced insulin responsiveness observed was correlated with increased insulin-induced Akt phosphorylation in muscle (Figure 4H) but not in the liver or adipose tissue (Figure S4D). In line with this, insulin-induced 2DG glucose uptake was significantly enhanced in the extensor digitorum longus (EDL) muscle isolated from *db/db* mice treated with BIBO3304 for 4 weeks, an effect that was impaired in the placebo group (Figure 4I). Strikingly, human muscle *NPY1R* expression was 3-fold higher in obese subjects compared with lean subjects (Figure 4J). The increased *NPY1R* expression in the human vastus lateralis muscle also exhibited a positive correlation with the BMI (Spearman's r = −0.6291, *P* = 0.005) as well as fasting blood glucose levels (Spearman's r = −0.5273, *P* = 0.0245) (Figure 4K and L). Consistently, we show in primary human myotubes that insulin-stimulated glucose uptake was suppressed significantly by NPY, an effect that was diminished in the presence of BIBO3304 (Figure 4M). Taken together, these results indicate that on the early onset of T2D, Y1 receptor antagonism attenuates hyperglycemia, which can be attributed to improved insulin action as a consequence of reduced adiposity and/or directly due to the inhibition of the Y1 receptor in muscle.

While BIBO3304 treatment of late-stage diabetic *db/db* mice at 16 weeks of age did not show any effects on body weights, lean mass, fat mass, fat pad mass, and insulin response (Figure S4E–I), and the impaired β-cell compensation in the 16-week-old *db/db* mice became evident as indicated by a greater than 2.5-fold reduction in the serum insulin level as compared with 10-week-old *db/db* mice (11.4 ± 1.91 ng/ml in 10-week-old vs 4.1 ± 0.26 ng/ml in 16-week-old *db/db* mice, *n* = 5–6) (Figure 4N). Interestingly, BIBO3304 treatment in 16-week-old *db/db* mice led to a significant enhancement of insulin secretion in response to re-feeding after an overnight fast when compared with the placebo group (Figure 4N), suggesting an increase in postprandial-induced insulin secretion. To further investigate whether Y1 receptor antagonism also has an effect on preserving β-cell mass during the transition from β-cell compensation to failure, pancreas from 16-week-old *db/db* mice treated daily for 6 weeks with BIBO3304 or placebo were examined. Pancreatic histological analysis revealed that pancreatic weights, pancreatic islet area, islet number, and islet proportion were comparable between the BIBO3304- and placebo-treated groups (Figure S4J–M). However, a significant increase in the intensity of insulin staining in β cells was observed in the pancreas from BIBO3304-treated *db/db* mice compared with placebo-treated *db/db* mice (Figure 4O). Collectively, these results suggest that Y1 antagonism preserves insulin action during early-stage diabetes while improving β-cell secretory capacity postprandially during late-stage diabetes, thereby improving the diabetic condition in *db/db* mice.

## 3 Discussion

In this study, we demonstrated that the observed increase in *NPY* and *NPY1R* mRNA expression in human islets was associated with reduced insulin secretion in human T2D. In addition, pharmacological inhibition of Y1 receptor signaling under diabetogenic conditions resulted in improved glucose-stimulated insulin secretion and reduced β-cell death in islets. Selective antagonism of the peripheral Y1 receptor with BIBO3304 enhanced β-cell function and preserved functional β-cell mass, thereby resulting in improved glycemic control in HFD/STZ-induced diabetic mouse models. Furthermore, BIBO3304 treatment on hyperinsulinemic *db/db* mice aged 4 weeks old resulted in reduced adiposity accompanied by lower fasting and postprandial blood glucose levels due to enhanced insulin signaling and glucose uptake in the skeletal muscle. Importantly, we also showed that the administration of BIBO3304 in diabetic *db/db* mice, whereby advanced β-cell decompensation is present, ameliorated diabetic condition by preserving functional β-cell mass during the transition from β-cell compensation to failure. These findings extend our previous studies which revealed that inhibition of Y1 receptor signaling improves β-cell function in both rodent and human islets, which can be utilized to improve islet transplantation outcomes and a delayed onset of T1D [16].

Compared with the critical role of neuronal NPY and its receptor Y1 in the regulation of appetite and energy metabolism, the role of NPY-Y1 receptor signaling in β-cell function and survival, particularly in human T2D, is far less clear. The present study is the first to report that augmented islet *NPY* and *NPY1R* mRNA expression from human subjects with T2D is associated with decreased islet insulin secretory capacity. Although previous studies on mice with global depletion of PYY-secreting cells [25] and β-cell-specific PYY transgenic mice [26] have revealed that islet-released PYY may act as a positive regulator of insulin secretion, it is important to note that these results were derived from mouse models with the supraphysiological phenotype. Therefore, the observed changes in insulin secretion may be secondary to the wide range of metabolic disturbances. Indeed, our findings in human islets are in line with previous studies revealing that increased islet *Npy1r* mRNA levels were present in diabetic mice induced by STZ [13,27,28] and in aged *db/db* mice with full-blown diabetes featuring impaired insulin secretion due to dedifferentiated β cells [11]. We further showed that increased islet Y1 signaling by selective Y1 agonists (e.g., [Leu^31^, Pro^34^] NPY) leads to impaired insulin secretion from isolated islets. Moreover, studies conducted on β-cell-specific *Npy* transgenic mouse models also reported that increased β-cell NPY/Y1 receptor signaling led to reduced islet glucose responsiveness [29]; in contrast, NPY/Y1 deficiency in mouse islet β cells enhanced glucose-induced insulin secretion *in vivo* [12,17]. Several lines of evidence have revealed that the elevated NPY-Y1 signaling in β cells can be triggered by oxidative stress [11], ER stress [30], and inflammation [31] in both mouse and human islets, therefore contributing to the impaired glucose responsiveness preceding diabetes. Indeed, failure to suppress NPY expression in β cells during metabolic stress was identified to be crucial in accelerating the programming of cell death [28]. Intriguingly, previous studies reported that treatment of Y receptor agonists, such as PYY administration, appears to protect islet β cells from apoptosis and dedifferentiation triggered by STZ in diabetic mice [[13], [14], [15],^32^]. Considering that the administration of PYY in diabetic mice induced significant weight loss, the mechanism underlying PYY's β-cell protective effects is likely attributable to its anorexic effect acting via the Y2 receptor rather than via the Y1 receptor on the hypothalamus. In contrast, the present study provides direct evidence, both *in vivo* and *ex vivo*, that pharmacological inhibition of the peripheral Y1 receptor by BIBO3304 can protect β cells against dysfunction and attenuate β cell death under various diabetogenic conditions. A previous study by Imai et al. reported that suppression of islet NPY and Y1 receptor mRNA expressions was associated with increased islet insulin secretion in HFD-fed C57BL/6 mice and leptin-deficient *ob/ob* mice [12], thus linking the reduced islet NPY-Y1 signaling to the natural course of β-cell compensation during obesity. Although increasing evidence suggested that hyperinsulinemia and diabetes remission in obese subjects post bariatric surgery are associated with increased serum PYY and GLP-1 levels, in addition to the substantial weight loss and reduced food intake resulting from decreased gastric capacity, the action of PYY on the hypothalamic Y2 receptor may also provide some beneficial effects [33]. However, in the context of islet function, the direct effect of increased PYY after bariatric surgery remains to be explored [34]. Collectively, these results demonstrate the significance of NPY-Y1 receptor signaling inhibition, which not only preserves insulin secretion but also protects β cells against apoptosis in T2D. Y1 receptors are G-protein-coupled receptors that are preferentially associated with G_i/o_ G-protein and therefore act in an inhibitory manner [35]. Intracellular cAMP levels are reduced in target cells in response to Y1 receptor ligands, whereas cAMP is increased in response to Y1 antagonism [36] as well as in islets isolated from Y1 receptor knockout mice [16]. The cAMP signaling-dependent mechanisms, mediated by cAMP-response element-binding protein (CREB), have been identified to play a critical role in improving insulin secretion and β-cell survival in diabetes [37]. Indeed, in addition to the changes in NPY/Y1 receptor expression, CREB activity in β cells was reportedly decreased under inflammation and ER stress, which leads to defects involving CREB-mediated anti-apoptotic gene expression, such as *bcl-2* and *xbp-1* [38]. In support of this notion, our findings demonstrated that *db/db* or WT islets treated with BIBO3304 were more resistant to stress-induced cell death under the conditions of elevated inflammatory cytokines, ER stress, and oxidative stress, whereby increase in NPY-Y1 signaling or decline in CREB activity was identified in islet β cells. The increased pCREB levels in *db/db* islets treated with BIBO3304 indicate that these protective effects are potentially attributable to the increased intracellular cAMP-CREB signaling, resulting from Y1 receptor antagonism. However, the impaired β-cell function induced by palmitate and H_2_O_2_ resulted in attenuated Ca^2+^ channel [39], indicating that the cAMP-dependent mechanisms remained intact, thus resulting in no further enhancement being observed under Y1 receptor antagonism. Together, these results indicate that dysregulated NPY/Y1 signaling in β cells acts as one of the key drivers to diabetes progression, as evident by the improved β-cell function under blockage of Y1 signaling.

Inhibition of Y1 receptors or NPY deficiency in the brain has been linked with decreased body weight gain and adiposity caused by suppression of the energy intake and increase in the energy expenditure [40,41]. Our findings revealed that the administration of the non-brain-penetrable Y1 receptor antagonist BIBO3304 also resulted in decreased body weight and fat mass with improved insulin response in *db/db* mice independent of the alterations in food intake, suggesting that the inhibition of peripheral Y1 receptor signaling can reduce adiposity by acting on mechanisms other than regulation of appetite centrally. This is consistent with a previous study conducted by Zhang et al. where conditional knockdown of Y1 receptors in the periphery exhibited reduced RER, indicating increased lipid oxidation [42]. Mechanisms underlying the increased lipid oxidation under peripheral Y1 antagonism were reported to be associated with increased carnitine palmitoyltransferase-1 (CPT-1) and upregulation of key enzymes involved in β-oxidation, consequently increasing the capacity of mitochondrial lipid oxidation and fatty acid transportation, particularly in the liver and muscle [42]. Interestingly, a recent study by Yan et al. demonstrated that the oral administration of BIBO3304 enhances thermogenesis through its effect on brown adipose tissue in diet-induced obese mice [22], although our study does not identify any noticeable change in Akt activity in adipose tissues in response to BIBO3304. However, in addition to reduced adiposity, BIBO3304 treatment in *db/db* mice also significantly enhanced insulin responsiveness as demonstrated by increased insulin-induced AKT phosphorylation and insulin-stimulated glucose uptake in the skeletal muscle of *db/db* mice and in primary human muscle cells. The insulin sensitizing effect observed in *db/db* mice might be, at least in part, due to reduced body weight and adiposity or muscle fat content. In line with our finding in primary human myotubes, previous studies showed that deficiency of the peripheral Y1 receptor results in increased mitochondrial capacity in the muscle [42], supporting a role of the Y1 receptor antagonism acting directly on muscle insulin receptor signaling. Nonetheless, these results are consistent with the notion that increasing muscle glucose uptake improves glycemic control and suggesting that, in addition to reduced adiposity, these effects may at least in part be responsible for the observed improvement in glucose homeostasis in BIBO3304-treated *db/db* mice.

In summary, one unmet clinical need in treating T2D is the availability of therapeutics that improves glycemic control by targeting the underlying β-cell dysfunction and failure. Our results revealed the dual effect of peripheral-specific Y1 receptor antagonism as a potential therapeutic strategy for T2D: 1) during the early pathogenesis of diabetes, whereby compensatory β-cell function is present driven by obesity and insulin resistance, we found that the inhibition of peripheral Y1 receptor signaling led to decreased adiposity, increased insulin sensitivity and, to a lesser extent, enhanced insulin-stimulated glucose uptake in the skeletal muscle. 2) During the advanced stage of T2D, where diabetic mice presented with β-cell failure, pharmacological inhibition of Y1 receptor preserved functional β-cell mass by protecting against β-cell death and improving β-cell function. As such, the reduced adiposity and improved insulin action coupled with the ability to preserve functional β-cell mass observed under the inhibition of NPY/Y1 signaling *in vivo* highlights a potential therapy for targeting peripheral Y1 receptor pathways, which may ultimately provide greater therapeutic benefits in controlling glucose levels in T2D.

## 4 Materials and methods

**Table:** Table 1. Key resources table

Reagent or resource	Source	Identifier
**Antibodies**		
Mouse monoclonal anti-ATK (pan) (40D4)	Cell Signaling Technology	Cat#: 2920S
Rabbit monoclonal anti-phospho-AKT (Ser 473)	Cell Signaling Technology	Cat#: 9271S
Rabbit monoclonal anti β-Actin	Cell Signaling Technology	Cat #: 4970; RRID: AB_2223172
Mouse monoclonal anti α-tubulin (DM1A)	Cell Signaling Technology	Cat#: 3873
Rabbit monoclonal anti-cleaved caspase-3 (Asp175) (5A1)	Cell Signaling Technology	Cat #: 9664S
Rabbit monoclonal anti-BIM (C34C5)	Cell Signaling Technology	Cat #: 2933T
Rabbit monoclonal anti-phospho-CREB (Ser133) (87G3)	Cell Signaling Technology	Cat #: 9198
Mouse mAb anti-CREB (86B10)	Cell Signaling Technology	Cat #: 9104
goat anti-rabbit-HRP antibody	Agilent Technologies	Cat#: P0448
Goat anti-mouse immunoglobulins/HRP	Agilent Technologies	Cat#:P0447
Guinea Pig Polyclonal Anti-Insulin	Agilent Technologies	Cat #: IR00261-2
Alexa Fluor 488 Goat Anti-Guine Pig IgG (H + L)	Life Technologies	A-11073
Alexa Fluor 594 Goat anti-Rabbit IgG (H + L)	Life Technologies	A11012
**Chemicals, peptides, and recombinant proteins**		
RPMI-1640 cell culture media	Sigma-Aldrich	
Streptozotocin	Sigma-Aldrich	Cat #: S0130
1,1-Dimethylbiguanide hydrochloride (metformin)	Sigma-Aldrich	Cat #: D150959
BIBO3304 trifluoroacetate	Tocris	Cat #: 2412
Actrapid (human, insulin)	Novo Nordisk Pharmaceuticals	Cat #:169625
**Critical commercial assays**		
Mouse insulin ELISA	ALPCO Diagnostics	Cat#: AP80-INSMS-E10
Glucose oxidase assay	ThermoFisher	Cat#: A22189
**Experimental models: organisms/strains**		
C57BL/6J		C57BL/6J
B6.BKS(D)Lepr ^db^/J (*db/db*)		*db/db*
**Oligonucleotides**		
*NPY* TaqMan® Gene Expression assay	ThermoFisher	Cat #: Hs00173470_m1
*PYY* TaqMan® Gene Expression assay	ThermoFisher	Cat #: Hs00373890_g1
*PPY* TaqMan® Gene Expression assay	ThermoFisher	Cat #: Hs00358111_g1
*NPY1R* TaqMan® Gene Expression assay	ThermoFisher	Cat #: Hs00702150_s1
*NPY2R* TaqMan® Gene Expression assay	ThermoFisher	Cat #: Hs01921296_s1
*PPYR1* TaqMan® Gene Expression assay	ThermoFisher	Cat #: Hs00275980_s1
*NPY5R* TaqMan® Gene Expression assay	ThermoFisher	Cat #: Hs01883189_s1
*RPLP0* TaqMan® Gene Expression assay	ThermoFisher	Cat #: Hs99999902_m1
*GAPDH* TaqMan® Gene Expression assay	ThermoFisher	Cat #: Hs99999905_m1
*Ppia* TaqMan® Gene Expression assay	ThermoFisher	Cat #: Mm02342430_g1
*Npy* TaqMan® Gene Expression assay	ThermoFisher	Cat #: Mm01410146_m1
*Pyy* TaqMan® Gene Expression assay	ThermoFisher	Cat #: Mm00520716_g1
*Npy1r* TaqMan® Gene Expression assay	ThermoFisher	Cat #: Mm00650798_g1
*Bak1* TaqMan® Gene Expression assay	ThermoFisher	Cat #: Mm00432045_m1
*Bax* TaqMan® Gene Expression assay	ThermoFisher	Cat #: Mm00432051_m1
*Bid* TaqMan® Gene Expression assay	ThermoFisher	Cat #: Mm00432073_m1
*Casp3* TaqMan® Gene Expression assay	ThermoFisher	Cat #: Mm01195085_m1
*Ddit3* TaqMan® Gene Expression assay	ThermoFisher	Cat #: Mm01135937_g1
AHLIKIN, Lepr TaqMan SNP assay	ThermoFisher	Cat #: 4332077
**Software and algorithms**		
Prism 8.0	Graphpad	https://www.graphpad.com/scientific-software/prism/
Image J	GNU General Public License	

### 4.1 Resource availability

#### 4.1.1 Lead contact

Further information and requests for reagents may be directed to lead author Dr Chieh-Hsin Yang (jyang@svi.edu.au).

#### 4.1.2 Materials availability

This study did not generate new unique reagents.

#### 4.1.3 Data and code availability

This study did not generate any unique datasets or code.

### 4.2 Experimental models and subject details

We obtained approval for performing human islet studies from St Vincent's Institute of Medical Research and St. Vincent's Clinical School Human Research Ethics Committee. Consent for the use of the islets for research was given by the relatives of the donors. Mice care and experiments were performed in accordance with the protocols approved by the Animal Ethics Committee at St Vincent's Hospital (AEC No. GBNML 760 and 016/19). Eight-week-old mice were fed a standard chow diet (6% fat) or a HFD (23% calories from fat; SF04-027; Specialty Feeds) as indicated. B6.BKS(D)Lepr ^db^/J (*db/+*) heterozygous mice were kindly provided by A/Prof Ross Laybutt from Garvan Institute of Medical Research (Sydney, NSW, Australia). Routine genotyping for homozygous *db/db* mice was conducted using TaqMan SNP genotyping assay (AHLIKIN, ThermoFisher Scientific). The eight-week-old C57BL/6 male mice were purchased from The Walter and Eliza Hall Institute (Victoria, Australia) for all *in vivo* studies. To induce T2D, C57BL/6 mice were fed a HFD (SF04-027; Specialty Feeds) for 4 weeks from 8 weeks of age, followed by multiple intraperitoneal injections of low-dose STZ (35 mg/kg) (Sigma Aldrich). STZ was prepared fresh each time in 0.1 M sodium citrate buffer (pH 4.5) and filter sterilized prior to use. The blood glucose level was measured twice a week until it reached 15 mmol/L and above. C57BL/6 mice used in islet experiments were bred in-house at BioResources Centre (St Vincent's Hospital). All mice were housed in a temperature-controlled room of 22 °C in a 12 h/12 h light/dark cycle (lights on from 07:00–19:00 h) with free access to water and food.

### 4.3 Method details

#### 4.3.1 Treatment with the Y1 receptor antagonist BIBO3304 and metformin

A non-brain-penetrable Y1 receptor antagonist BIBO3304 (Tocris Bioscience) was prepared in Milli Q water at a concentration of 1 mg/ml. C57BL/6 mice (average weight 27.4 ± 0.3 g) were administered BIBO3304 daily in jelly containing 4.9% (wt/v) gelatine and 7.5% (v/v) chocolate flavoring essence at a dose of 0.5 μmol/mouse/day (an estimated dose of 0.02 mg/kg), as described previously [16]. The obese *db/db* mice at 4 (average weight 20.3 ± 0.9 g) or 12 weeks of age (average weight 42 ± 1.3 g) were given BIBO3304 once daily via oral gavage at a dose of 2.5 μmol/mouse/day, while control mice on placebo treatment received the same volume of Milli Q water. Metformin (Sigma Aldrich) was prepared in Milli Q water, and 0.25 g/kg was administered daily via oral gavage. The duration of treatment is as stated in the text for each procedure.

#### 4.3.2 Metabolic assessment and body composition measures

The effects of the Y1 receptor antagonist BIBO3304 on blood glucose control and body weight were monitored weekly on the same day of the week between 09:00 h and 10:00 h. RBG levels were measured on an Accu-Check Performa glucometer (Roche, Switzerland) using blood collected by the tail tipping method. For the fast-refeeding experiment, food was removed from the mice at the dark cycle before the experiment. Blood was collected by retro-orbital bleed after a 16-h fast as well as 30 min after refeeding to determine blood glucose and plasma insulin levels. Plasma glucagon levels were assessed in samples collected under feeding conditions and assayed by a mouse glucagon ELISA kit (Crystal Chem Inc., USA). Food intake was measured at the same time points (n = 8 per group). Whole body lean mass and fat mass were measured at the end of the study using the whole-body composition analyzer, EchoMRI (Houston, USA).

#### 4.3.3 *In vivo* assessment of glucose, insulin, and pyruvate tolerance tests

Glucose tolerance tests were performed on 6-h-fasted HFD/STZ mice or overnight-fasted *db/db* mice by intraperitoneal injection of 1 g/kg and 0.5 g/kg glucose, respectively. Insulin tolerance was measured by intraperitoneal injection of 0.75 i.u./kg and 2.5 i.u./kg human insulin (Actrapid, Novo Nordisk Pharmaceuticals) on HFD/STZ mice and *db/db* mice after a 6-h fast. Pyruvate tolerance tests were conducted on mice after an overnight fast with intraperitoneal injection of 1 g/kg sodium pyruvate. Blood glucose was measured at basal and 15, 30, 60, 90, and 120 min following glucose, insulin, or pyruvate administration. The *in vivo* GSIS was determined by the intravenous glucose tolerance test using 1 g/kg glucose on overnight-fasted HFD/STZ mice. Briefly, the mice were anesthetized, and jugular venous catheters were inserted. The mice were allowed to recover for 20 min after surgery. A bolus of glucose was given via a catheter and blood glucose was measured at 2, 5, 10, 15, and 30 min post glucose administration.

#### 4.3.4 Pancreatic islet isolation and culture *ex vivo*

Mouse islets were isolated from C57BL/6 and *db/db* mice, as described previously [43]. Briefly, collagenase P (0.45 mg/ml) (Sigma Aldrich) was injected into the bile duct to distend the pancreas. After perfusion, the pancreas were excised and incubated at 37 °C for 15 min. The islets was further purified using the Histopaque-1077 gradient (Sigma Aldrich). The isolated mouse islets were cultured in Connaught Medical Research Laboratories (CMRL) 1066 medium (Invitrogen, Life Technologies, Carlsbad, CA, USA) supplemented with 10% fetal calf serum, 100 U/ml penicillin, 100 mg/ml streptomycin, and 2 mmol/L l-glutamine. The isolated islets were incubated in a 37 °C, 5% CO_2_ humidified incubator.

#### 4.3.5 Human islet isolation and culture *ex vivo*

The pancreases were obtained from heart-beating, brain-dead donors with consent from next-of-kin and research approval from the St Vincent's Hospital, Melbourne (HREC-011-04). Human islets were purified using Ficoll density gradients [44] and cultured in CMRL 1066 medium (Invitrogen, Life Technologies, Carlsbad, CA, USA) supplemented with 10% fetal calf serum, 100 U/ml penicillin, 100 mg/ml streptomycin, and 2 mmol/L l-glutamine. All islets were incubated in a 37 °C, 5% CO_2_ humidified incubator. The insulin stimulation index was determined and presented as the ratio of insulin secretion at 28 mmol/L to that of at 2.8 mmol/L from the same islets.

#### 4.3.6 Skeletal muscle biopsies from human donors

Eighteen nondiabetic males with an average of 40.4 ± 3.9 years of age, body mass index (BMI) 27.7 ± 1.4 kg/m^2^, and fasting blood glucose of 4.9 ± 0.15 mmol/L were included. Muscle samples were acquired from the *vastus lateralis* under local anesthesia (Xylocaine 1%) using the percutaneous needle biopsy technique with suction. The samples were snap frozen in liquid nitrogen and stored at −80 °C until analyses. The methods for participant recruitment and muscle biopsy were approved by the Human Research Ethics Committee, Victoria University.

#### 4.3.7 GSIS in isolated islets

Wild-type C57BL/6 islets were incubated in the respective diabetogenic stressors for the indicative duration: Inflammation: islets were incubated with proinflammatory cytokine cocktail (50 ng/ml TNFa, 250 ng/ml IFNg, and 25 ng/ml IL1b) for 48 h; oxidative stress: 10 mM H_2_O_2_ for 16 h; ER stress: 1 mM thapsigargin for 24 h; glucolipotoxicity: 500 μM palmitate + 25 mM glucose for 48 h. Following culture, the islets were handpicked and pre-incubated for 1 h in HEPES-buffered-KREBS buffer containing 0.2% BSA and 2.8 mmol/L d-glucose. Subsequently, 15 size-matched islets were incubated at 37 °C for another 1 h in KREBS buffer containing either 2.8 mmol/L or 20 mmol/L d-glucose and treated with or without 1 μM BIBO3304. The culture medium was collected, and insulin secretion was assayed using a mouse insulin ELISA kit (ALPCO Diagnostics, Salem, NH, USA).

#### 4.3.8 DNA fragmentation assay

To induce islet cell death, wild-type C57BL/6 islets were incubated in the respective diabetogenic stressors: Inflammation: islets were incubated with proinflammatory cytokine cocktail (50 ng/ml TNFa, 250 ng/ml IFNg, and 25 ng/ml IL1b) for 72 h; oxidative stress: 70 μM H_2_O_2_ for 18 h; ER stress: 5 mM thapsigargin for 72 h; glucolipotoxicity: 25 mmol/L glucose plus 0.5 mM palmitate for 96 h. Cell apoptosis was measured by analysis of DNA fragmentation, as described previously [45]. Briefly, islets of uniform size were handpicked into 3.5 cm Petri dishes containing the appropriate stimuli to induce apoptosis in 1.5 ml complete CMRL medium. At the end of the culture period, the islets were dispersed by trypsin digestion for 5 min at 37 °C, followed by mechanical disruption by pipetting up and down for 10 times. The dispersed islet cells were then resuspended in 150 ml of Nicoletti buffer containing 50 mg/ml propidium iodide (Miltenyi Biotec), 0.1% (wt/v) sodium citrate, and 0.1% (v/v) Triton X-100 [46]. The cells were then analyzed on a LSRFortessa Flow Cytometer (Becton Dickinson, Franklin Lakes, NJ). Cells undergoing apoptosis were identified by their apparent sub-diploid DNA content as reported previously [47].

#### 4.3.9 2-Deoxyglucose uptake measurement in the EDL muscle

Db/db mice were fasted overnight and then euthanized in a CO_2_ chamber. Left and right EDL muscles were quickly excised and bathed in carbogenated Krebs-Henseleit buffer (KHB) (119 mM NaCl, 4.7 mM KCl, 2.5 mM CaCl_2_, 1.2 mM MgSO_4_, 1.2 mM KH_2_PO_4_, 25 mM NaHCO_3_, pH 7.4, 30 °C) with constant shaking. After 30 min of pre-incubation, the muscles were transferred to fresh carbogenated KHB containing 10 mU/mL insulin (or KHB without insulin as control) for 30 min. Subsequently, the muscles were transferred to fresh KHB containing 2 mM 2-deoxy-d-[1,2-3H]-glucose (0.15 μCi/mL) and 16 mM d-[1-^14^C] mannitol (0.1 μCi/mL) for 15 min. After the incubation, the muscles were rapidly rinsed with ice-cold KHB buffer, then snap frozen in liquid nitrogen and stored at −80 °C. Subsequently, the muscle samples were lysed in ice-cold radioimmunoprecipitation assay (RIPA) buffer (400 μl/muscle) with protease and phosphatase inhibitor cocktail (Cell Signaling) using TissueLyser II (QIAGEN). Half of the lysate was mixed with scintillation cocktail for scintillation counting using a liquid scintillation analyzer (PerkinElmer), and the other half was used for immunoblotting.

#### 4.3.10 2-Deoxyglucose uptake measurement in human myotubes

Three lines of primary human myoblasts originating from the skeletal muscle samples of three nondiabetic male participants (age: 64, 72, and 80 years) were used to assess insulin-stimulated glucose uptake. Myogenic differentiation of the myoblasts was initiated when the cells grew to ∼80% confluence in 12-well plates, the growth medium (10% fetal bovine serum [FBS] in α-MEM) was replaced with the differentiation medium containing 2% horse serum in α-MEM. After 5 days of differentiation (the differentiation medium was refreshed every other day), the cells were treated with/without 0.5 μM NPY and/or 1 μM BIBO3304 in serum-free medium for 24 h. Following the treatment, the cells were stimulated with 100 nM insulin (or without insulin) in glucose uptake buffer (GUB) (10 mM HEPES, 2.5 mM NaH_2_PO_4_, 150 mM NaCl, 5 mM KCl, 1.2 mM CaCl_2_, 1.2 mM MgSO_4_, 0.1% BSA, pH 7.4) for 45 min. In the last 15 min of stimulation, 1 mM 2-deoxy-d-[1,2-^3^H]-glucose (1 μCi/mL) was spiked into the GUB. After the incubation, the cells were washed three times with ice-cold PBS and then lysed with 0.1 M NaOH (200 μl/well). 150 μl of the lysate was pipetted into vials with scintillation cocktail for scintillation counting, and the remaining lysate was used in protein assay for normalization.

#### 4.3.11 RNA extraction and quantitative real-time PCR

Total RNA of the mouse islets was extracted using an RNeasy Plus Mini Kit (Qiagen). Other tissues including mouse liver, muscle, and adipose tissues were excised and snap frozen in liquid nitrogen, and RNA was isolated using RNAzol reagent (Sigma, St. Louis, MO) following the manufacturer's instructions. Isolated mRNA was reverse transcribed into cDNA using the Superscript IV First-Strand Synthesis System (Invitrogen, Australia), and quantitative real-time PCR was performed using the Light-Cycler 480 Real-Time PCR system (Roche, Switzerland). The relative gene expression of NPY ligands and receptors was performed under the assumption that the binding efficiency of the probes are equal. *RPLP0* and *GAPDH* were used as the housekeeping gene for the normalization of NPY ligands and receptors in human islets and human muscle, respectively. *Ppia* was used as a housekeeping gene for the normalization of NPY and NPY1 receptors in mouse islets. Primer details are listed in the Supplementary Table 2. The amplification conditions used in all the RT-qPCR experiments were as follows: 95 °C for 10 min, 95 °C for 15 s, and 60 °C for 60 s for 40 cycles. Relative quantification was determined using the 2^−ΔΔCt^ method.

#### 4.3.12 Immunoblotting

A total of 200 islets per sample from *db/db* or *db/+* mice were cultured in 3 ml of complete CMRL medium and treated with or without BIBO3304 (1 μM) for 36 h. The islets were lysed in ice-cold RIPA lysis buffer supplemented with protease and phosphatase inhibitor cocktails (Cell Signaling Technology). The protein concentrations were determined with BCA protein assay (Pierce, Thermo Fisher Scientific). Proteins were resolved in SDS-PAGE gel (4–20% gradient polyacrylamide gel electrophoresis, Mini-PROTEAN® Precast Gels, Biorad). Blots were blocked for 1 h with 5% nonfat dry milk in PBS/0.1% tween-20 (Sigma Aldrich) and subsequently incubated overnight at 4 °C with the respective primary antibodies: pan-AKT antibody (40D4) (1:2,000; 2920S; Cell Signaling Technology), phospho-AKT (Ser473) (1:1,000; 9271S; Cell Signaling Technology), BIM (C34C5) (1:1,000; 2933T; Cell Signaling Technology), cleaved caspas-3 (Asp175) (5A1E) (1:1,000; 9664S; Cell Signaling Technology), phosphor-CREB (Ser133) (1:1000; 9198S; Cell Signaling Technology), β-actin (1:2,000; 4970; Cell Signaling Technology), or a-tubulin (1:1,000; 3873; Cell Signaling Technology). Following incubation, the membranes were washed for 3 × 10 min in PBS-T and then incubated with HRP-linked secondary antibodies for 1 h in 5% milk in PBS-T at room temperature. After washing for 3 × 10 min, the immunoreactive signals were visualized using the SuperSignal™ West Femto Maximum Sensitivity Substrate (Thermo Fisher Scientific) and then developed using the Super RX Fuji X-ray film (Fujifilm, Tokyo Japan). Protein band intensities were quantified using Image J. Cleaved caspase-3 and BIM protein signals were normalized against tubulin as a loading control.

#### 4.3.13 Immunofluorescent staining on pancreatic histochemical analysis

Whole pancreas was excised and fixed in 4% PBS-buffered paraformaldehyde overnight at room temperature and embedded into paraffin. Slides with 5-μm-thick pancreatic sections were deparaffinized using histolene (Trajan Scientific, Australia), rehydrated with ethanol (100%, 100%, 90%, 70%), and blocked in 10% FBS in PBS for 30 min at room temperature. Subsequently, the sections were incubated for 2 h at room temperature with polyclonal guinea pig anti-insulin antibody (1:5, Agilent Technologies). The slides were then washed 3 × 5 min with PBS and incubated with the anti-guinea pig IgG Alexa Fluor 488 (1:200, Life Technologies) diluted in 10% FBS for 1 h at room temperature. The resulting slides were then mounted in a mounting medium containing DAPI. The slides were scanned at 20× magnification using a 3D Histech Panoramic SCAN II slide scanner (Phenomics Australia Histopathology and Slide Scanning Service, University of Melbourne). For β-cell mass measurement, islets were outlined manually on the digital images. The islet area and islet number were analyzed using digital image processing software Image Scope (Aperio). Two sections separated by at least 150 μm were used for each mouse (*n* = 8 per treatment). The cell mass of pancreatic β-cells was determined as the product of wet pancreatic weight and the ratio of insulin-positive/total pancreatic area.

#### 4.3.14 Hepatic glucose production assay

The primary hepatocytes were isolated from C57BL/6 mice at 8 weeks of age and plated at a density of 1 × 10^6^ cells in 6-well plates with the plating medium (Williams' E medium supplemented with 10% FBS, 1% penicillin–streptomycin, and 1% of l-glutamine) for 4 h followed by starvation overnight in low-glucose DMEM-supplemented 1% l-glutamine and 1% penicillin–streptomycin. The following day, the cells were pretreated with/without 0.5 mM NPY and/or 1 mM BIBO3304 for 1 h. Subsequently, the cells were washed once with PBS, and the assay medium (DMEM without glucose, 1% penicillin–streptomycin, 2 mM of sodium pyruvate, and 20 mM sodium lactate, pH 7.4) was added with/without 0.5 mM NPY and/or 1 mM BIBO3304 for 6 h. Glucose production was assayed with the Amplex Red glucose assay kit (Invitrogen), and cell lysate was used in protein assay for normalization.

#### 4.3.15 Statistical analysis

All data are presented as mean ± SEM. A Student's t-test was conducted to test the differences between the two groups of mice. Restricted randomization was used to achieve similar numbers of mice in each treatment group. The sample size was estimated based on previously published studies by us and by other research groups [16,17,48,49]. The differences among the groups of mice were assessed by two-way ANOVA or repeated-measures ANOVA. The correlation coefficient was calculated using Spearman's rank correlation coefficient. Statistical analyses were performed using Prism software 8.0. All experiments requiring the use of animals or primary hepatocytes or islets were subject to randomization based on litter. The differences were regarded as statistically significant if ∗*P* < 0.05, ∗∗*P* < 0.01 and, ∗∗∗*P* < 0.001.

## Author contributions

C.H.Y, D.A.O, X.Z.L, S.N, S.F, and E.P designed and performed research and contributed to discussion, C.H.Y, J.W.S, J.O, S.G, and Y.S contributed to discussion and reviewed the manuscript. A.M-A and C.S contributed to the research experiments and reviewed the manuscript. T.L, I.L, R.D.L, and H.H contributed to discussion and edited the manuscript. H.E.T and K.L contributed to the discussion, wrote the manuscript, and reviewed/edited the manuscript. All authors read and approved the final manuscript.
