# Supplementary material for: Neuropeptide Y1 receptor antagonism protects β-cells and improves glycemic control in type 2 diabetes
Source: Mol Metab. 2021 Dec 7;55:101413. doi: 10.1016/j.molmet.2021.101413 (PMC8733231; doi:10.1016/j.molmet.2021.101413)
Supplement: Multimedia component 1 [file mmc1.docx]

Supplementary Table 2

| Oligonucleotides |  |  |
| --- | --- | --- |
| *NPY* TaqMan® Gene Expression Assay | ThermoFisher | Cat #: Hs00173470_m1 |
| *PYY* TaqMan® Gene Expression Assay | ThermoFisher | Cat #: Hs00373890_g1 |
| *PPY* TaqMan® Gene Expression Assay | ThermoFisher | Cat #: Hs00358111_g1 |
| *NPY1R* TaqMan® Gene Expression Assay | ThermoFisher | Cat #: Hs00702150_s1 |
| *NPY2R* TaqMan® Gene Expression Assay | ThermoFisher | Cat #: Hs01921296_s1 |
| *PPYR1* TaqMan® Gene Expression Assay | ThermoFisher | Cat #: Hs00275980_s1 |
| *NPY5R* TaqMan® Gene Expression Assay | ThermoFisher | Cat #: Hs01883189_s1 |
| *RPLP0* TaqMan® Gene Expression Assay | ThermoFisher | Cat #: Hs99999902_m1 |
| *GAPDH* TaqMan® Gene Expression Assay | ThermoFisher | Cat #: Hs99999905_m1 |
| *Ppia* TaqMan® Gene Expression Assay | ThermoFisher | Cat #: Mm02342430_g1 |
| *Npy* TaqMan® Gene Expression Assay | ThermoFisher | Cat #: Mm01410146_m1 |
| *Pyy* TaqMan® Gene Expression Assay | ThermoFisher | Cat #: Mm00520716_g1 |
| *Npy1r* TaqMan® Gene Expression Assay | ThermoFisher | Cat #: Mm00650798_g1 |
| *Bak1* TaqMan® Gene Expression Assay | ThermoFisher | Cat #: Mm00432045_m1 |
| *Bax* TaqMan® Gene Expression Assay | ThermoFisher | Cat #: [Mm00432051_m1](https://www.thermofisher.com/taqman-gene-expression/product/Mm00432051_m1?CID=&ICID=&subtype=) |
| *Bid* TaqMan® Gene Expression Assay | ThermoFisher | Cat #: [Mm00432073_m1](https://www.thermofisher.com/taqman-gene-expression/product/Mm00432073_m1?CID=&ICID=&subtype=) |
| *Casp3* TaqMan® Gene Expression Assay | ThermoFisher | Cat #: Mm01195085_m1 |
| *Ddit3* TaqMan® Gene Expression Assay | ThermoFisher | Cat #: Mm01135937_g1 |
| AHLIKIN, Lepr TaqMan SNP Assay | ThermoFisher | Cat #: 4332077 |
